# Pituitary macroadenoma with persisting dense lymphocytic infiltration in a young male patient 

**DOI:** 10.5414/NP300378

**Published:** 2011-10-18

**Authors:** E. Cakir, C. Bayindir, P.A. Sabanci, M. Imer, N.C. Ozbey

**Affiliations:** 1 Department of Pathology,; 2 Department of Neurosurgery; 3 Department of Medicine, Division of Endocrinology, Istanbul University, Istanbul Medical Faculty, Istanbul, Turkey

**Keywords:** pituitary adenoma, hypophysitis, transsphenoidal surgery, prolactin

## Abstract

Context: Lymphocytic anterior hypophysitis in association with a pituitary adenoma was reported previously. In rare instance, inflammatory infiltration was confined to adenoma tissue itself, excluding anterior pituitary. Case: The patient – a 27-year-old male – presented with visual field defect. Further examination revealed a pituitary mass with suprasellar extension. Hormonal evaluation indicated mild hyperprolactinemia (42 ng/ml, normal < 19). After transsphenoidal resection, a pituitary adenoma showing cytoplasmic immunoreactivity to prolactin was identified. Dense and diffuse lymphocytic infiltration was seen within the tumor. At 15th month, a second transsphenoidal operation was necessary because of rapid development of visual compromise and headache. Excised surgical specimen consistent with previously resected adenoma showed diffuse lymphocytic infiltration composed of B and T cells within the adenoma tissue again. Conclusion: Presence of dense, hypophysitis-like lymphocytic infiltration within pituitary adenoma tissue obtained by two consecutive operations may reflect an host-mediated immune reaction to tumor. This rare finding could be challenging in terms of differential diagnosis and follow-up course.

## Introduction 

Lymphocytic hypophysitis is a rare lesion leading to hypopituitarism and mass effects [1]. The disease shows a predominant female predilection of approximately 6 : 1, and young women during late pregnancy or in the postpartum period are commonly affected [1, 2]. Clinical presentation and radiological findings may mimic a pituitary macroadenoma [3]. Male patients with biopsy-proven lymphocytic hypophysitis with a preoperative diagnosis of pituitary adenoma are described also [4]. Lymphocytic hypophysitis in association with a pituitary macroadenoma is reported as a rare association. According to our knowledge five cases have been reported previously. First reported case was a 22-year-old female patient who presented with amenorrhea and galactorrhea. Histopathological examination revealed a clinically silent sparsely granulated growth hormone cell adenoma with lymphocytic infiltration of the adjacent pituitary tissue [5]. The second case was a 43-year-old female patient who presented with galactorrhea and hyperprolactinemia due to stalk compression. Histopathological examination revealed a nonsecreting pituitary adenoma with concurrent lymphocytic adenohypophysitis [6]. A non-functioning pituitary adenoma in association with lymphocytic hypophysitis was reported in a 38-year-old female patient [7]. The 2 other cases were a 39-year-old female patient and 61-year-old male patient with functioning pituitary adenomas in association with lymphocytic hypophysitis [8]. However in all of these cases, lymphocytic infiltration was seen within the nearby normal anterior pituitary tissue, thereby confirming the diagnosis of adenoma tissue associated lymphocytic adenohypophysitis. Inflammatory infiltration confined to adenoma tissue itself, excluding anterior pituitary was reported in a prolactinoma patient previously [9]. 

In this study, we present a young male patient with a pituitary macroadenoma showing cytoplasmic immunoreactivity to prolactin in association with dense and diffuse lymphocytic infiltration within the adenoma tissue and his follow-up course. 

## Case history 

**Figure 1.**
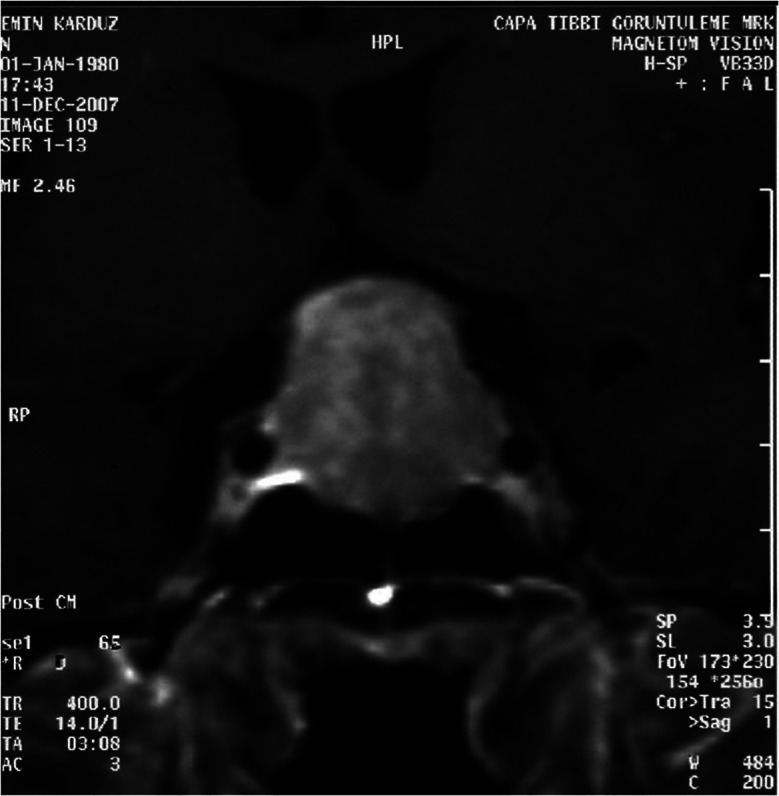
Preoperative sella MRI of the patient indicating a 3 × 2.8 × 2.2 cm intrasellar mass extending superiorly.

A 27-year-old male patient was admitted with partial vision loss and decreased libido. Neurological examination revealed bilateral temporal hemianopia. A cranial magnetic resonance imaging (MRI) scan was performed. A hypophyseal macroadenoma extending to suprasellar region was detected. The patient was referred to the neurosurgery clinic. Symptoms were present for 6 months. Decreased frequency of beard shaving was reported. The patient was married and had two children. The younger child was 11-month-old. Physical examination revealed normal blood pressure and pulse rate. Hormone profile revealed following findings; prolactin: 42.1 ng/ml (reference range (RR): 2.58 – 18.12), morning cortisol:12.4 µg/dl (RR: 5.0 – 25.0), luteinizing hormone (LH): 3.9 mlU/ml (RR: 1.8 – 8.16), follicle stimulating hormone (FSH): 4.4 mlU/ml (RR: 1.37 – 13.58), free testosterone: 8.24 pg/ml (RR: 8.9 – 42.5), testosterone: 3.0 ng/ml (RR: 2.8 – 8.0), thyroid stimulating hormone (TSH): 3.45 mIU/l (RR: 0.35 – 4.94), free T4: 15.3 pmol/l (RR: 12 – 22), T3: 3.16 pmol/l (RR: 2.63 – 5.7 pmol/l), insulin-like growth factor-1 (IGF-1): 94.2 ng/ml (RR: 117 – 329). Prolactin concentrations were measured after 1/100 dilution to eliminate hook effect. No change was observed. Therefore mild increase in prolactin was thought to be caused by stalk compression when the presence of a large macroadenoma was considered. No abnormality was seen in serum biochemical results. In “Visual Evoked Potential” (VEP) study, bilateral P-100 latency was lengthened. Optic neuropathy was then suspected. The patient’s new sella MRI revealed an intrasellar 3 × 2.8 × 2.2 cm mass that enhances homogenous contrast material after Gadolinium injection (Figure 1). 

**Figure 2.**
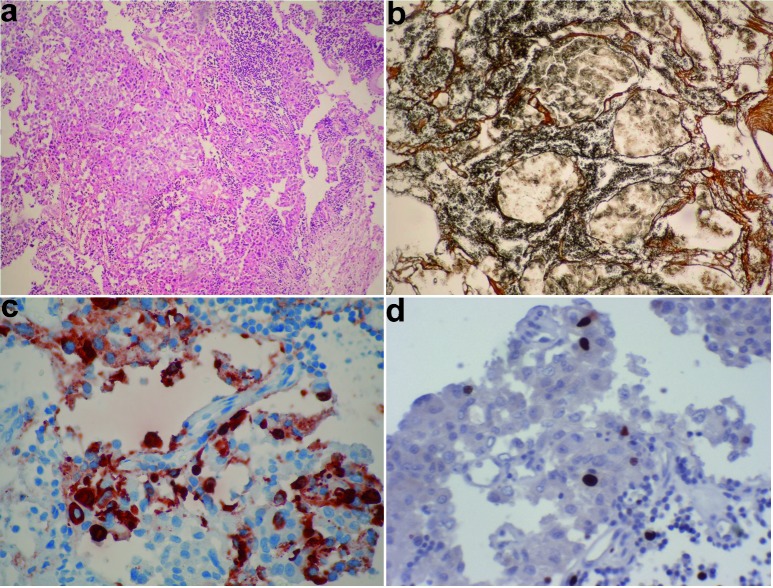
a: Adenoma tissue and lymphocytic infiltration within the tumoral tissue (HE × 100); b: Loss of normal acinar architecture within the adenoma tissue (Gomori-Silver × 100); c: Adenoma tissue showing cytoplasmic immunoreactivity to prolactin (Anti-prolactin monoclonal antibody × 400); d: Ki-67 staining indicating labeling index of 3% (× 200).

After endocrinology consultation, an urgent operation to relief optic chiasma compression was decided. The adenoma was resected subtotally via endonasal transsphenoidal approach. During the operation 3 samples were taken; the first from duramater, the second from gelatinous spontaneously draining adenomatous tissue, the third one from a solid lesion which was fixed to the sellar basement. Pathological examination revealed that all three samples were concordant with a pituitary adenoma. A dense and diffuse lymphocytic infiltration was seen within the adenoma tissue (Figure 2a). Normal aciner architecture was distorted (Figure 2b). Prolactin, TSH, growth hormone, LH, FSH and adrenocorticotopin hormone (ACTH) immunoreactivities were studied using monoclonal antibodies: anti-prolactin antibody (Thermo-Fischer Scientific, Rockford, IL, USA) diluted 1 : 400; anti-TSH antibody (BioGenex, San Ramon, CA, USA) diluted 1 : 100; anti-growth hormone antibody (BioGenex) diluted 1 : 100; anti-bLH antibody (Dako North America, Carpinteria, CA, USA) diluted 1 : 50; anti-bFSH antibody (BioGenex) diluted 1 : 100; anti-ACTH antibody (Dako North America) diluted 1 : 75. Normal anterior pituitary and null cell adenoma tissues were used as positive and negative controls for these antibodies respectively. Ki-67 labeling index was performed using a monoclonal antibody (Thermo-Fischer Scientific) diluted 1 : 200. 

Adenoma cells showed immunoreactivity to prolactin (Figure 2c). Intracytoplasmic staining for prolactin was observed in 50 – 60% of adenoma cells. LH, FSH, TSH, growth hormone and ACTH immunostainings were negative. Ki-67 labeling index was found as 3% (Figure 2d). A fibrous pseudo-capsule surrounded the adenoma. Cells of adenoma had large acidophilic cytoplasm and round-ovoid nucleus. No anterior pituitary tissue fragment was available for histopathological evaluation. 

**Figure 3.**
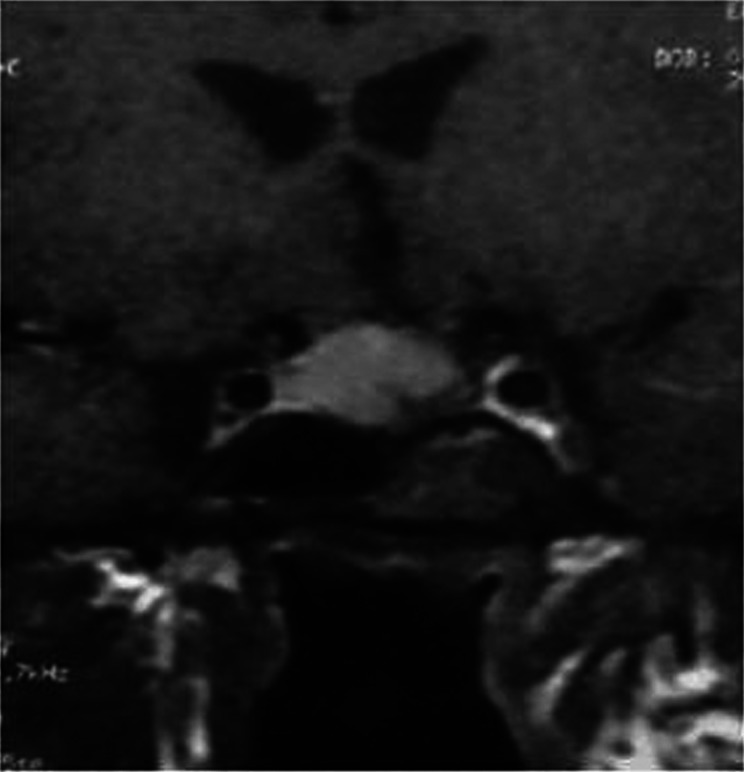
MRI of the sellar region at 4th month after the first operation.

After the operation, the patient’s vision improved markedly and objectively when preoperative and postoperative perimeter tests were compared. Postoperative pituitary function evaluation revealed hypogonadotropic hypogonadism and normal prolactin concentrations (prolactin: 11.8 and 10.9 ng/ml). Prolactin concentrations, measured several times after the operation, remained within normal range. Short ACTH stimulation test at the 3rd month revealed adequate cortisol reserve. The patient was given parenteral androgen replacement therapy after the operation. At the 4th month, control MRI showed resolution of the optic nerve compression and a decrease in the tumor volume (Figure 3). 

The patient was followed-up with serial visual field and symptoms evaluation while on testosterone replacement therapy. Sella MRI performed at 11th month postoperatively revealed no significant progression in the residual tissue. No new symptoms were evident. At 15th month, headache, fatigue and blurred vision developed. Visual field examination showed progression of visual field defect, in particular for the left eye, although radiologically significant progression of the mass lesion was not detected. Because of the progressive deterioration in vision, second surgery was planned. 

**Figure 4.**
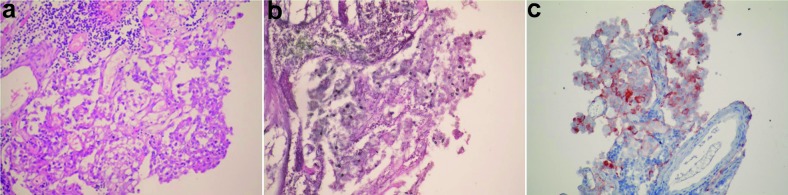
a: Adenoma tissue and lymphocytic infiltration within the tumoral tissue (HE × 100) (second operation material); b: Loss of acinar and reticular architecture within the adenoma tissue (Gomori-Silver × 100) (second operation material); c: Adenoma cells showing immunoreactivity to prolactin (Anti-prolactin monoclonal antibody × 200) (second operation material).

**Figure 5.**
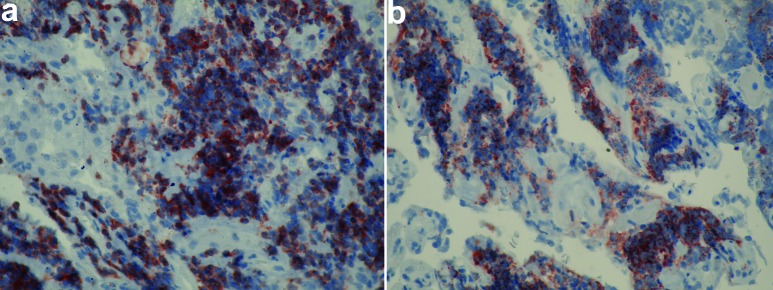
a: Diffuse lymphocytic infiltration, cells showing strong immunoreactivity to CD3 indicating presence of T lymphocytes (Anti-CD3 monoclonal antibody × 400) (second operation material); b: Diffuse lymphocytic infiltration, cells showing strong immunoreactivity to CD20 indicating presence of B lymphocytes (Anti-CD20 monoclonal antibody × 400) (second operation material).

The patient was operated again at the 15th month after his first operation via sublabial transsphenoidal approach and the recurrent tumor was resected. An adenoma tissue with diffuse lymphocytic infiltration and similar immunohistochemical characteristics of previously resected adenoma was seen (Figure 4a, b, c). Dense lymphocytic infiltration within the adenoma tissue was composed of both B and T cells (Figure 5a, b). CD68 immunostaining was negative. CD 68 immunoreactivity was studied using a monoclonal antibody (Thermo-Fischer Scientific) diluted 1 : 400. T and B cell immunoreactivities were determined using anti-CD3 and anti-CD20 monoclonal antibodies (NovoCastra, NewCastle, UK, diluted 1 : 200 and Neomarkers, Fremont, CA, USA, diluted 1/200 respectively). No apparent anterior pituitary tissue component was available for diagnostic evaluation. 

**Figure 6.**
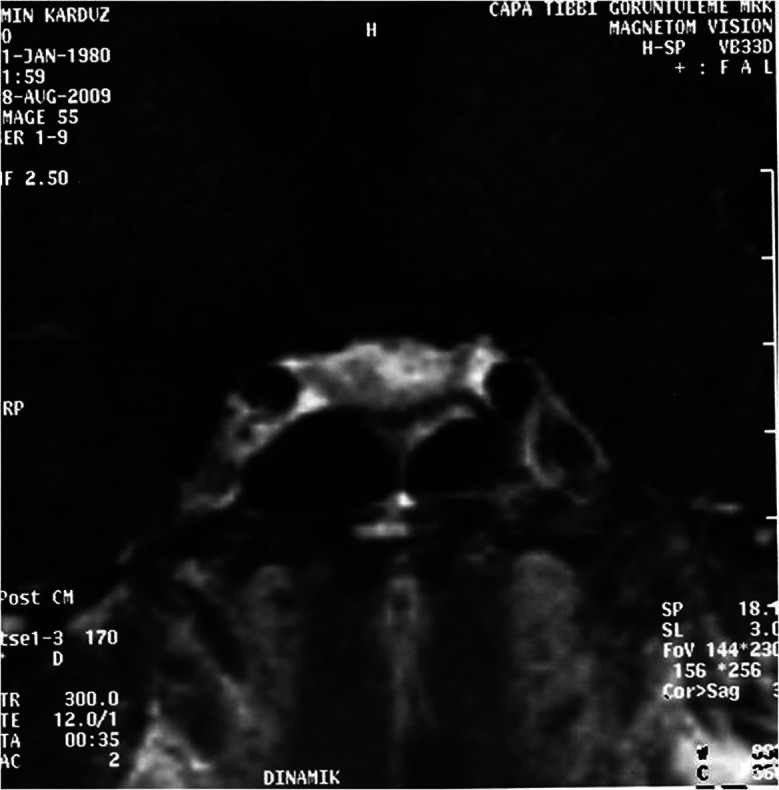
MRI of the sellar region after the second operation, showing postoperative changes.

After the operation, ACTH deficiency was added to gonadotropin deficiencies. On the last control MRI scan 4 months later, postoperative changes were detected (Figure 6). 

## Discussion 

Lymphocytic hypophysitis is suggested to have an autoimmune origin [2, 10]. Antipituitary antibodies which characterize pituitary autoimmunity and lymphocytic hypophysitis are also found after traumatic brain injury and postpartum pituitary necrosis as a secondary phenomenon [11, 12]. In addition, an inflammatory reaction involving adjacent tissue and/or cerebrospinal fluid secondary to intrapituitary tumors other than pituitary adenomas are described previously [13, 14]. The term of “secondary hypophysitis” is suggested to indicate the pituitary inflammation originated from adjacent lesions or as a part of systemic disease [15]. Secondary hypophysitis could be an inflammatory reaction to the released tumor/pituitary tissue antigens. Pituitary adenomas are reported to contain some degree of lymphocytic infiltration within the adenoma tissue in 2.9% of cases (40/1400 pituitary adenomas) [16]. In this study, infiltration patterns are found as perivascular (n = 22), interstitial (diffuse, n = 8; nodular, n = 5) or mixed (n = 2) and almost entirely composed of T cells [16]. Degree of lymphocytic infiltration is not reported [16]. Findings of the present study correspond to diffuse interstitial pattern. But both T and B lymphocytes are evident within the adenoma tissue. 

Vajtai et al. [9] reported histopathological findings of a prolactinoma and intratumoral infiltration by reactive lymphocytes. The patient was a 31-year-old female patient. Her preoperative prolactin concentration was 140 ng/ml. Dopamine agonists were not given and the patient underwent pituitary surgery for a 12 mm macroadenoma. A prolactin cell adenoma was found. An inflammatory infiltrate mostly composed of T cells, lesser amounts of B cells and scarse monocytes was observed along intra-tumoral vascular septa. Residual normal pituitary tissue was free of inflammatory cells. Vajtai et al. [9] suggested that those findings represented cell-mediated and a short-lived immune reaction directed at a subpopulation of tumor cell and would eventually subside. They also proposed that this transitory phenomenon could be missed since most prolactinomas came to surgery after a period of medical treatment and follow-up. However, follow-up course of the patient presented here revealed that, this inflammation may persist as long as 15 months. A more recent study by Lupi et al. [17], indicated 18 out of 72 pituitary adenoma showed mononuclear cell infiltration, assessed by CD 45; a cell surface marker of T lymphocytes. In this study 13 of 18 adenomas with T lymphocyte infiltration had a score of 1 or 0.5 indicating mild degree of T lymphocyte infiltration and remaining 5 cases showed moderate degree of infiltration (score 2 and 3). Lymphocytic hypophysitis gained a score of 5 according to this study and normal pituitary gland obtained at autopsy studies had a score of 0. According to this scoring system, the presented patient’s pituitary adenoma had a score of 5. Mononuclear cell infiltration was composed of both T and B cells. Therefore we think that the present case is unique by the presence of dense, hypophysitis-like infiltration of both T and B lymphocytes within adenoma tissue that persisted for 15 months. Lupi et al. [17] also indicated presence of T lymphocytes in the adenoma tissue whether hormone secreting or not, was an independent prognostic factor for persistence/recurrence of pituitary adenoma. The follow-up course of the patient presented here supported this conclusion. 

We cannot totally exclude the involvement of pituitary adenoma by coexistent lymphocytic adenohypophysitis of anterior pituitary in our patient. But this occurrence seems highly unlikely when previous cases of hypophysitis in association with a pituitary adenoma are considered. In those cases [5, 6, 7, 8] inflammatory infiltrate did not primarily involve tumoral tissue. In one of the recent cases, pituitary mass enlargement during follow-up, leading to transsphenoidal operation was detected. It was suggested that enlargement of the pituitary lesion could not be caused by preexisting tumor enlargement, but lymphocytic hypophysitis of the nonneoplastic tissue developed during follow-up could be responsible for the increased pituitary mass size [7]. In the present case, we suggest that the progression in inflammatory infiltration may lead to rapid development of clinical symptoms. 

Pro-inflammatory cytokines and/or antigenic fragments released from tumoral tissue could provoke a diffuse inflammatory reaction within the tumor [6, 8, 9, 16, 17]. Presence of B lymphocytes in the present case may indicate local antibody production against tumor antigens. Positive immunostaining for prolactin may play a paracrine role for this inflammatory infiltration. In Heshmati et al.’s study [16], lymphocytic infiltrate is significantly more frequent in prolactin-staining adenomas than in immune-negative adenomas. A close correlation is evident between prolactin concentrations and autoimmune activity [18]. We suggest that presence of dense, hypophysitis-like lymphocytic infiltration within the pituitary adenoma persisting in two consecutive operations reflects an host-mediated immune reaction to tumor. Further studies are needed to determine different and unique immunological behavior of pituitary adenomas. In the present case, no clinical and laboratory findings indicating systemic inflammatory syndromes were evident until this time. In addition, thyroid antibodies, gastric parietal cell antibody and anti-nuclear antibody tests were negative. 

Prolactin concentrations were not high enough to suggest a macroprolactinoma in the present study, although positive prolactin immunostaining was evident within the adenoma cells’ cytoplasm. A rough correlation is evident between prolactin secreting adenoma size and prolactin concentrations. A pituitary prolactin secreting adenoma > 1 cm is frequently associated with serum prolactin concentrations > 250 ng/ml [19]. Some pituitary adenomas do not secrete and lead to a clinical hormone hypersecretion syndrome, although hormone immunoreactivity is found within the cell cytoplasm [20]. In a small percentage of clinically non-functioning pituitary adenomas, positive immunoreactivity to ACTH, growth hormone, prolactin or TSH (singly or combination) are observed [21, 22]. 

## Conclusion 

The patient presented here has an unusual finding: having dense and diffuse hypophysitis-like lymphocytic infiltration within a pituitary adenoma which persisted as long as 15 months and confirmed by two consecutive operations. Presence of dense, hypophysitis-like lymphocytic infiltration within pituitary adenoma tissue obtained by two consecutive operations may reflect a host-mediated immune reaction to tumor.****
